# BRAF Alterations in Chronic Lymphocytic Leukemia: Genomic Landscape, Co-Mutation Patterns, and Clinical Relevance

**DOI:** 10.1007/s11899-026-00780-7

**Published:** 2026-05-01

**Authors:** Abdulrahman Al-Abdulmalek, Eric Landry, Mohammed Abdulgayoom, Abdulrahman F. Al-Mashdali, Ayman Abdullah Dalol, Bassam Muthanna, Jean-Pierre Routy, Shehab F. Mohamed

**Affiliations:** 1 Division of Hematology, McGill University Health Centre, Montreal, QC H4A 3J1 Canada; 2 Faculty of Medicine and Health Sciences, McGill University, Montreal, QC H4A 3J1 Canada; 3 Department of Hematology, National Center for Cancer Care and Research, Doha, Qatar; 4 College of Medicine, Qatar University, Doha, Qatar; 5 Internal Medicine Department, University of Missouri, Columbia, Missour 65211 USA; 6 Research Institute of the McGill University Health Centre, Montreal, QC H4A 3J1 Canada

**Keywords:** Chronic lymphocytic leukemia, BRAF mutations, MAPK pathway, Richter transformation, Clonal evolution, Targeted therapy resistance

## Abstract

**Purpose of Review:**

BRAF alterations are uncommon in chronic lymphocytic leukemia (CLL), yet increasing use of broad genomic profiling has identified them as a recurrent component of MAPK-pathway dysregulation. This revised review summarizes the reported frequency, mutation spectrum, co-mutation patterns, treatment-era associations, and clinical implications of BRAF alterations in adult CLL, with explicit separation of chemoimmunotherapy-dominant cohorts from targeted-therapy-era cohorts.

**Recent Findings:**

Across published studies, BRAF mutations are usually detected in approximately 2–6% of unselected CLL cohorts, with higher frequencies in biologically enriched or treatment-selected populations. In chemoimmunotherapy-dominant cohorts, the clinically relevant signals are more often seen in treatment-timing endpoints such as time to first treatment, treatment-free survival, or time to next treatment than in overall survival alone. In targeted-therapy-era studies, including phase II and real-world cohorts, BRAF alterations recur as part of MAPK-pathway–driven clonal evolution at relapse after BTK-, PI3K-, or BCL2-directed therapy. Small pathology-based series also suggest that BRAF V600E is more frequent in Richter transformation than in untransformed CLL. By contrast, direct evidence for BRAF inhibitors in CLL is very limited, and available preclinical data do not support routine single-agent BRAF inhibition for the predominantly non-V600E lesions seen in CLL.

**Summary:**

Current evidence supports interpreting BRAF-mutated CLL within the broader RAS–RAF–MAPK–ERK signaling context rather than as a classical V600E-driven entity. At present, BRAF is best viewed as a biologic and resistance-relevant annotation rather than an established standalone prognostic biomarker or routine therapeutic target. Its clinical relevance appears greatest in trisomy 12-enriched disease, genomically complex cases, Richter transformation with V600E lesions, and treatment-exposed relapse where pathway-directed strategies may become increasingly important.

## Introduction

Chronic lymphocytic leukemia (CLL) is a biologically and clinically heterogeneous disease, with trajectories ranging from prolonged indolent courses to rapidly progressive disease requiring early treatment. This variability is shaped by recurrent genomic lesions involving genes such as TP53, NOTCH1, SF3B1, ATM, XPO1, and BIRC3, as well as cytogenetic abnormalities including del(17p), del(11q), trisomy 12, and del(13q), together with differences in IGHV mutational status, all of which influence disease biology and clinical outcome [1]. Although the major genomic drivers of CLL have been well characterized, alterations affecting the RAS–RAF–MAPK signaling pathway have received comparatively less attention.

The RAS–RAF–MAPK–ERK pathway plays a central role in regulating cell proliferation, survival, and differentiation, and activating lesions in this pathway are well recognized in several hematologic malignancies. BRAF V600E is a defining lesion in hairy cell leukemia, present in the vast majority of cases [2], and recurrent BRAF alterations have also been described in histiocytic disorders such as Langerhans cell histiocytosis [3]. In CLL, however, BRAF mutations appear to be less common and biologically distinct from the canonical oncogenic driver pattern seen in these other diseases.

Over the past decade, multiple sequencing and translational studies have identified BRAF mutations as a recurrent, albeit uncommon, genomic event in CLL [4–19]. Early screening studies detected these alterations at low frequency, often through assays focused on the V600E hotspot [16, 19]. Later genomic analyses using broader sequencing platforms demonstrated a wider mutational spectrum and suggested that non-V600E variants predominate in CLL, supporting a disease-specific biological context for BRAF activation [12, 13, 15, 18]. More recent treatment-era studies have further shown that BRAF alterations may arise within a broader cooperative genomic framework, either co-occurring with other driver lesions or emerging during clonal evolution. In particular, therapy-exposed investigations have linked MAPK pathway alterations to disease progression or resistance during BTK inhibitor, PI3K inhibitor, and venetoclax-based treatment [4–6, 9, 14].

The clinical relevance of BRAF in CLL appears to depend on the treatment context. In untreated or chemoimmunotherapy-era cohorts, BRAF alterations have been linked mainly to earlier need for treatment or shorter treatment-free intervals, while a consistent adverse effect on overall survival has not been shown [10, 13, 15]. In contrast, in patients relapsing after targeted therapies, BRAF is probably more informative as a marker of clonal evolution and MAPK-pathway–mediated resistance than as a baseline prognostic factor. In this setting, its importance lies in suggesting relapse biology and supporting interest in pathway-directed or combination treatment strategies [4, 6, 9, 14].

To clarify the available evidence, we performed a narrative review of published studies reporting BRAF mutations or MAPK-pathway alterations in adult CLL, focusing on mutation frequency, variant spectrum, co-mutation patterns, era-specific outcome associations, Richter transformation, and therapeutic implications. Given the marked heterogeneity in study populations, detection methods, treatment exposure, and endpoint reporting, the evidence was synthesized descriptively rather than quantitatively.

## Study Characteristics and Era-Based Framework

Overall, the datasets included were published between 2012 and 2025 and were dominated by retrospective cohort studies and translational genomic analyses, with later incorporation of phase II and phase III correlative cohorts [4–19]. The geographic footprint was largely European and international, with strong representation from Spain, Germany/Switzerland, Italy, Ireland, the United Kingdom, the United States, and multicenter international targeted-therapy studies [4–19]. Where demographic data were reported, the median age generally clustered in the sixth to seventh decades, and most cohorts showed a male predominance consistent with the epidemiology of CLL [4–19].

To improve interpretability, we stratified the literature into two clinically meaningful groups: (1) chemoimmunotherapy-dominant or untreated/mixed baseline cohorts, in which BRAF is mainly examined as a biologic or prognostic variable [7, 8, 10–13, 15–19], and (2) targeted-therapy-era, therapy-exposed cohorts, in which BRAF more often appears as part of clonal evolution or resistance biology [4–6, 9, 14].

From a technical standpoint, targeted next-generation sequencing was the most commonly used detection platform in recent studies [4–8, 10, 11, 13], whereas earlier reports relied on V600E-specific assays, Sanger sequencing, whole-exome sequencing, or whole-genome sequencing [12, 14–19]. Tables 1A and B summarize the included datasets according to clinical era/context.

**Table 1:** Published cohorts reporting BRAF alterations in chronic lymphocytic leukemia, stratified by treatment context.

Study	Setting	Design	Population	BRAF tested	BRAF-mutated *n* (%)	Predominant type	Detection method
A chemoimmunotherapy-dominant, untreated, or mixed baseline cohorts reporting BRAF alterations in CLL							
Langabeer et al., [19]	Ireland	Brief report/screening cohort	CLL/PLL/SLL; CLL subset screened for BRAF V600E	151	1 (0.66%)	V600E only	AS-PCR + Sanger
Jebaraj et al., [18]	Germany/Switzerland	Retrospective cohort + in vitro	CLL including unselected and fludarabine-refractory cohorts	138	4 (2.90%)	Mostly non-V600E	Direct Sanger sequencing
Damm et al., [17]	France/Japan collaboration	Translational molecular study	CLL discovery and validation cohorts	192	8 (4.17%)	Non-V600E favored	WES + targeted Sanger
Sellar et al., [16]	Multinational	Observational screening study	CLL/Richter syndrome screened for BRAF V600E	302	7 (2.32%)	V600E only	VE1 IHC → ASO-PCR/Sanger
Pandzic et al., (Spain cohort) [15]	Spain	Retrospective cohort within functional study	Unselected CLL cohort	506	10 (1.98%)	Mostly non-V600E	WES/WGS
Pandzic et al., (UK CLL4 cohort) [15]	United Kingdom	Retrospective trial-based cohort	Previously untreated CLL from CLL4 trial	247	8 (3.24%)	Mostly non-V600E	Targeted NGS
Vendramini et al., [13]	Italy/USA	Retrospective cohort	Treatment-naïve CLL enriched for trisomy 12	534	24 (4.49%)	Predominantly non-V600E	NGS
Giménez et al., [12]	Spain	Retrospective cohort	ICGC-CLL cohort	452	9 (1.99%)	All reported as non-V600E	WGS/WES
Hernández-Sánchez et al., [11]	Spain	Retrospective cohort	Hyperdiploid CLL and parallel unselected controls	57	2 (3.51% overall; 28.6% in hyperdiploid subgroup)	Non-V600E	Targeted NGS
Pérez-Carretero et al., [10]	Spain	Retrospective cohort	Untreated IGH-translocated CLL subset	233	5 (2.15%)	Non-V600E only reported	Targeted NGS
Pérez-Carretero et al., [8]	Spain	Retrospective cohort	del-3′IGH CLL genomics subset	54	4 (7.41%)	Subtype not fully detailed	Targeted NGS
Lütge et al., [7]	Germany/Switzerland	Translational genomic profiling	Typical/atypical CLL, mixed treated and untreated	157	9 (5.73%)	Subtype not detailed	Targeted sequencing/WES/WGS
B. Targeted-therapy-era, therapy-exposed cohorts reporting BRAF alterations in CLL							
Herling et al., [14]	Germany/Australia	Prospective translational cohort	Relapsed/refractory TP53-aberrant CLL on venetoclax	8	1 (12.5%)	K601E reported	Whole-exome sequencing
Murali et al., [9]	USA	Exploratory translational study	Multiply relapsed CLL on PI3K inhibitors	28	5 (17.86%)	Mixed; non-V600E favored	Whole-exome sequencing
Bonfiglio et al., [6]	International multicenter	Retrospective real-world cohort	CLL relapsing on ibrutinib vs. ongoing responders	98	4 (4.08%)	Mixed/unclear	NGS panel
Jain et al., [5]	International multicenter	Phase II trial subanalysis	Previously untreated CLL/SLL relapsing after fixed-duration ibrutinib-venetoclax	190	12 (6.32%)	Subtype not detailed	Targeted DNA NGS
Brown et al., [4]	International multicenter	Phase III trial genomic subset	Relapsed/refractory CLL progressing on zanubrutinib or ibrutinib	52	10 (19.23%)	Subtype not detailed	High-sensitivity targeted NGS

*AS-PCR* allele-specific polymerase chain reaction, *CLL* chronic lymphocytic leukemia, *ICGC-CLL* International Cancer Genome Consortium for CLL, *IGH* immunoglobulin heavy chain, *NGS* next-generation sequencing, *PI3K* phosphoinositide 3-kinase, *PLL* prolymphocytic leukemia, *SLL* small lymphocytic lymphoma, *WES* whole-exome sequencing, *WGS* whole-genome sequencing.

## Frequency and Mutation Spectrum

Across the included studies, BRAF alterations were consistently rare but reproducible. In broad or mixed CLL cohorts, the prevalence usually fell between about 2% and 6%, with higher rates in selected settings such as progression cohorts, hyperdiploid CLL, trisomy 12-enriched disease, or molecularly selected relapse cohorts [8, 11–13, 15]. This variation is best explained by case-mix, treatment exposure, and assay breadth rather than by contradictory biology.

Non-V600E lesions predominate in CLL when broad sequencing is used, whereas V600E-only assays identify very few cases and therefore underestimate total BRAF-mutated disease burden [12, 13, 16, 18, 19]. When subtype-level detail was reported, the balance consistently favored non-V600E disease: Jebaraj et al. found three non-V600E and one V600E mutation among four BRAF-mutated CLL cases; Pandzic et al. reported nine of ten non-V600E variants in the Spanish cohort and seven of eight in the UK CLL4 cohort; Pérez-Carretero et al. identified only non-V600E lesions in the IGH-translocated subset; Hernández-Sánchez et al. found two of two non-V600E lesions in hyperdiploid CLL; Herling et al. described a K601E mutation during venetoclax resistance; Giménez et al. reported nine of nine non-V600E cases; and Vendramini et al. identified 24 predominantly non-V600E BRAF mutations in trisomy 12-enriched CLL [8, 10–15, 18]. By contrast, V600E was largely confined to isolated cases in early screening studies and to small Richter syndrome-focused observations [16, 19].

This distinction is important because BRAF alterations in CLL are biologically and clinically different from the classic BRAF V600E–driven pattern seen in diseases such as hairy cell leukemia. In CLL, most reported BRAF lesions are non-V600E variants, and their significance is usually shaped by the broader genomic background, prior treatment exposure, and the stage of disease at which they are detected [2, 4, 6, 9, 12–15, 18].

## Clinicogenomic Associations

**Fig. 1:**
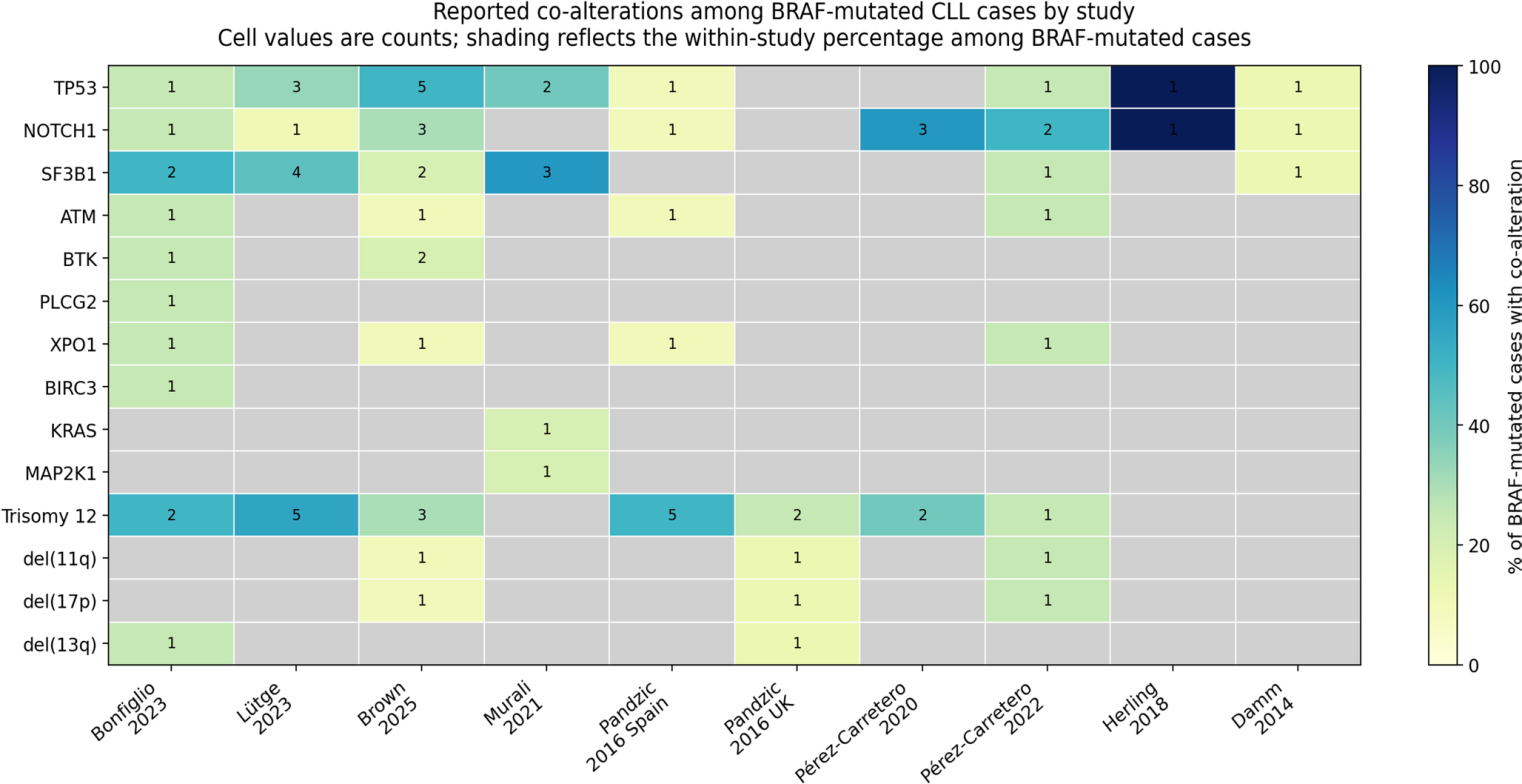
Co-mutation heat map for selected studies with study-level co-alteration data

Figure 1 shows the reported co-alterations among BRAF-mutated CLL cases across the included studies. The most commonly reported gene-level co-alterations were TP53, NOTCH1, and SF3B1, each identified in several independent cohorts. Additional co-alterations, including ATM, BTK, PLCG2, XPO1, BIRC3, KRAS, and MAP2K1, were reported less frequently and were more study-specific. Among cytogenetic abnormalities, trisomy 12 was the most recurrent co-occurring lesion, while del(11q), del(17p), and del(13q) were reported in a smaller number of studies [4, 6–8, 10–15].

The number and type of co-alterations varied across cohorts, and not all studies assessed the same genomic or cytogenetic markers. As such, the figure summarizes the available reported co-alterations by study rather than implying a single uniform genomic signature [4, 6–15].

## Era-Epecific Clinical Associations and Outcomes

Only a limited number of studies reported direct BRAF-mutated versus BRAF wild-type comparisons, and most involved very small numbers of altered cases. Nevertheless, once the literature is separated by clinical era, a useful pattern emerges: in chemoimmunotherapy-dominant cohorts, treatment-timing endpoints such as time to first treatment, treatment-free survival, or time to next treatment are often more informative than overall survival alone [10, 12, 13, 15, 20].

In the untreated IGH-translocated subset described by Pérez-Carretero et al. [10], BRAF-mutated cases had a markedly shorter median time to first treatment than wild-type cases (2 versus 23 months), whereas progression-free survival did not differ significantly. Similarly, pathway-level analyses by Giménez et al. [12] linked RAS-BRAF-MAPK-ERK mutations to worse 5-year time to first treatment and an independent multivariable hazard ratio for earlier treatment, while isolated overall-survival differences were less convincing. By contrast, the two Pandzic cohorts did not show an overall-survival difference according to BRAF status [15]. A large randomized-trial analysis from the UK LRF CLL4 program further supports the broader relevance of MAPK-ERK lesions in chemotherapy-era disease, although that study was pathway-level rather than BRAF-specific [20].

Targeted-therapy-era studies are clinically informative in a different way. Rather than demonstrating a stable baseline prognostic effect, these studies show repeated emergence or persistence of BRAF/MAPK lesions at relapse after targeted therapy. Herling et al. identified a BRAF K601E lesion during venetoclax resistance evolution [14]. Murali et al. linked MAPK-pathway activation to PI3K-inhibitor resistance [9]. Bonfiglio et al. reported additional non-BTK/non-PLCG2 genomic mechanisms in a multicenter real-world ibrutinib-relapse cohort that included BRAF alterations [6]. Jain et al. extended this concept to relapse after fixed-duration ibrutinib-venetoclax in a phase II cohort [5], and Brown et al. identified acquired BRAF lesions among patients progressing in the ALPINE study [4]. Taken together, these data indicate that in the targeted era, BRAF is most meaningful as a marker of pathway-level escape and clonal evolution [4–6, 9, 14].

Because outcome reporting was inconsistent across studies, particularly for treatment-anchored endpoints, we prioritized time to first treatment, treatment-free survival, and relapse-context findings where available; however, these findings should be interpreted cautiously [10, 12, 13, 15, 20] Table 2.

**Table 2:** Outcome studies relevant to BRAF-mutated or MAPK-pathway–altered CLL

Study/cohort	Molecular subset	Evaluable altered cases (*n*)	Comparator cases (*n*)	Endpoint(s)	Key result	Interpretation
Pérez-Carretero et al., [10]	BRAF-mutated within IGH-translocated CLL	5	43	TTFT/PFS	Median TTFT 2 vs. 23 months (*P* = 0.042); median PFS 39 vs. 24 months (*P* = 0.53)	Suggests earlier treatment need in a biologically selected untreated subgroup.
Pandzic et al., (Spain cohort) [15]	BRAF-mutated unselected CLL	10	496	OS	Median OS 128.0 vs. 122.9 months (*P* = 0.98)	No clear OS difference detected.
Pandzic et al., (UK CLL4 cohort) [15]	BRAF-mutated previously untreated trial cohort	8	239	OS	Median OS 79.0 vs. 75.0 months (*P* = 0.97)	No clear OS difference detected.
Giménez et al., [12]	RAS-BRAF-MAPK-ERK pathway-mutated CLL (pathway-level, not BRAF-isolated)	25	427	TTFT/OS	Worse 5-year TTFT; multivariable HR for TTFT 1.8; OS not independently adverse without co-high-risk lesions	Treatment-timing signal was stronger than OS, supporting pathway-level clinical relevance.
Herling et al., [14]	BRAF K601E in TP53-aberrant relapsed/refractory CLL on venetoclax	1	7	Response/relapse	The BRAF-mutated case achieved a partial response and then relapsed	Supports clonal evolution during venetoclax exposure rather than a validated prognostic effect.

*CLL* chronic lymphocytic leukemia, *OS* overall survival, *PFS* progression-free survival, *TTFT* time to first treatment, *TTNT/TNT* time to next treatment, *IGH* immunoglobulin heavy chain

## Discussion

Several practical conclusions emerge from the literature. First, BRAF-mutated CLL is uncommon but real, and the mutation spectrum is fundamentally different from what clinicians may expect from hairy cell leukemia or melanoma. In other hematologic malignancies, BRAF often behaves as a more canonical driver lesion: BRAF V600E is a defining hallmark of hairy cell leukemia and a major MAPK lesion in Langerhans cell histiocytosis and related histiocytic neoplasms [2, 3]. CLL is different. The predominance of non-V600E lesions is one of the most reproducible findings across broad sequencing studies and should shape both interpretation and therapeutic speculation [12–14, 16, 18].

###  Cytogenetics and Co-Mutations

At the cytogenetic level, trisomy 12 emerged as the most repeatedly reported chromosomal abnormality co-occurring with BRAF mutations. This finding is consistent with prior observations that RAS–BRAF–MAPK pathway lesions are enriched in trisomy 12 CLL, supporting the concept that BRAF-mutated CLL may preferentially cluster within a distinct biologic subgroup. By contrast, del(11q), del(17p), and del(13q) were reported less often and appeared more unevenly across studies [10–13, 15].

Another important observation from Fig. 1 is the marked between-study heterogeneity in both the number and pattern of co-alterations. Some studies reported only a limited number of recurrent partners, whereas others demonstrated broader co-mutational complexity. This likely reflects both biologic diversity and methodological differences. In particular, studies using broader next-generation sequencing panels or whole-exome approaches tended to identify a wider range of co-alterations than earlier targeted studies. Likewise, cohorts composed of relapsed, refractory, or progression-phase patients appeared more likely to show multiple concurrent abnormalities than treatment-naïve cohorts, consistent with clonal evolution under therapeutic pressure [4, 6, 7, 9, 14].

Taken together, these findings suggest that BRAF-mutated CLL is best understood as part of a broader cooperative genomic framework, rather than as a stand-alone lesion. The recurrent co-occurrence with TP53, NOTCH1, SF3B1, and trisomy 12 supports the view that BRAF mutations may mark a biologically distinct subset characterized by genomic complexity, signaling activation, and potentially more aggressive disease behavior. However, the currently available evidence remains largely study-level and descriptive. Therefore, these co-alteration patterns should be interpreted as hypothesis-generating rather than definitive evidence of a uniform molecular signature [10, 12, 13].

###  Prognostic Implications and Clinical Impact

The prognostic significance of BRAF alterations in CLL remains uncertain. Only a small number of studies directly compared outcomes between BRAF-mutated and BRAF-wild-type cases, and in most of these studies the number of BRAF-mutated patients was very limited. As a result, robust comparative estimates were generally not available, and most outcome data were descriptive [10, 12, 14, 15].

Overall, the available evidence does not support BRAF as an established independent prognostic marker in CLL. Instead, BRAF alterations seem to occur more often in biologically higher-risk settings, including trisomy 12-enriched disease, IGH-associated genomic subgroups, MAPK-pathway–activated disease, and therapy-exposed relapse. When clinical associations were reported, they more often involved earlier need for treatment or shorter treatment-free intervals than a consistent adverse effect on overall survival. This suggests that treatment-timing endpoints, such as time to first treatment, treatment-free survival, or time to next treatment, may better reflect the clinical relevance of BRAF than overall survival, although these outcomes were not uniformly reported across studies [4–6, 9, 10, 12, 13, 13, 14, 14, 15, 20].

Taken together, the most balanced interpretation is that BRAF is not currently suitable for routine risk stratification in CLL. Rather, it is better viewed as a marker of adverse biological context in selected subsets. Its main clinical value may lie in interpretation alongside co-mutation profile, cytogenetic background, and disease phase, particularly when detected at relapse or progression on targeted therapy [4, 6, 9, 10, 12, 13, 13, 14, 14, 15].

###  BRAF in Treatment-Exposed Disease and Resistance

Studies from the targeted-therapy era suggest that the significance of BRAF in CLL lies less in baseline prevalence and more in its role at relapse. BRAF alterations have been reported in patients progressing on BTK inhibitors, PI3K inhibitors, and venetoclax-based regimens, often together with other signaling abnormalities rather than as isolated lesions [4–6, 9, 14]. This pattern supports the idea that BRAF participates in clonal evolution and pathway-level escape under treatment pressure, rather than acting as a single dominant resistance mechanism.

Murali et al. provided important mechanistic support for this concept by showing that MAPK-pathway activation can mediate resistance to PI3K inhibition in relapsed CLL [9]. Likewise, Bonfiglio et al. and Brown et al. identified BRAF alterations in patients progressing on BTK inhibitors, including some cases without detectable BTK or PLCG2 mutations [4, 6]. Herling et al. described a non-V600E BRAF lesion during venetoclax resistance evolution [14], while Jain et al. showed that relapse after fixed-duration ibrutinib-venetoclax may occur even in the absence of canonical BTK, BCL2, or PLCG2 mutations [5]. Together, these observations suggest that relapse in CLL cannot always be explained by the usual resistance mutations alone and that MAPK-pathway activation may represent an alternative route of disease escape in a subset of patients.

In the venetoclax setting, the most appropriate conclusion is therefore not that BRAF-mutated CLL is uniformly resistant to BCL2-directed therapy, but that MAPK-pathway lesions may contribute to relapse biology in selected cases. This also provides a biologic rationale for dual-targeted strategies, such as BTK inhibitor plus BCL2 inhibitor combinations, which may better suppress parallel survival pathways and reduce dependence on any single escape route. However, this remains a pathway-level, hypothesis-generating interpretation rather than evidence for a BRAF-specific treatment recommendation [5, 14].

### Richter Transformation and Why this Review Matters

A possible association between BRAF and Richter transformation has been reported, although the evidence remains limited. The strongest support comes from a small pathology-based study in which BRAF V600E was identified more often in Richter syndrome than in untransformed CLL [16]. This finding is important because it contrasts with the broader CLL literature, where non-V600E variants predominate. As a result, V600E-positive Richter-transformed disease should not be interpreted as representative of BRAF-mutated CLL as a whole.

This distinction also clarifies the clinical relevance of the review. The importance of BRAF in CLL lies less in its overall frequency and more in whether it identifies biologically meaningful subsets at important clinical transitions, including earlier treatment need, targeted-therapy relapse, and transformation. For this reason, BRAF remains relevant to contemporary precision-based interpretation of CLL, even if it does not yet have a routine role in frontline risk stratification [4–6, 10, 12, 14, 16].

###  What is Known About BRAF-Directed Therapy?

Direct evidence for BRAF inhibitors in CLL is very limited, and the currently available data do not support routine extrapolation from melanoma or hairy cell leukemia. Jebaraj et al. reported limited evidence of cell-death induction with BRAF inhibition in primary CLL cells [18]. More detailed functional work by Giménez et al. showed that vemurafenib did not meaningfully inhibit ERK phosphorylation in primary MAPK-pathway–mutated CLL cells, while dabrafenib showed only modest and partly nonspecific effects; in contrast, the downstream ERK inhibitor ulixertinib more effectively suppressed phospho-ERK in mutated samples [12].

These findings fit the underlying biology: most CLL-associated BRAF lesions are non-V600E and are embedded within broader signaling networks. Moreover, paradoxical ERK activation with BRAF inhibition has been described in CLL-relevant settings, reinforcing caution about indiscriminate use of single-agent BRAF inhibitors [21]. The one context in which BRAF-directed therapy may be more plausible is transformed, V600E-positive Richter syndrome, but even there the evidence remains limited to small case-based or pathology-based observations rather than prospective CLL trials [12, 16, 21].

## Strengths of This Review

A major strength of this review is its effort to place BRAF alterations in CLL within their proper clinical and biologic context. The available literature is limited and heterogeneous, but this review synthesizes it in a way that distinguishes early-disease cohorts from targeted-therapy relapse cohorts, where the relevance of BRAF is fundamentally different. By integrating mutation spectrum, co-alteration patterns, treatment exposure, and Richter-related observations, the review moves beyond simple prevalence estimates and offers a more clinically meaningful interpretation of when BRAF may matter in CLL.

##  Limitations

Several limitations should be acknowledged. First, the available literature on BRAF alterations in CLL remains limited, and most studies report very small numbers of BRAF-mutated cases, restricting the ability to draw definitive conclusions regarding prognosis or treatment response. Second, substantial methodological heterogeneity exists across studies, including differences in sequencing platforms, mutation-detection sensitivity, and genomic panel breadth, which may influence reported mutation frequencies and subtype distributions. Third, many cohorts differ in clinical context, ranging from untreated populations to heavily pretreated or progression-phase disease, complicating direct comparison across eras. Fourth, treatment-timing endpoints such as TTNT/TNT were not consistently reported in BRAF-specific series, making cross-study outcome synthesis imperfect. Finally, some earlier investigations relied on V600E-specific assays, which likely underestimated the true prevalence of the non-V600E variants now recognized to predominate in CLL.

##  Future Directions

Future work should focus on defining when BRAF alterations in CLL are biologically informative and when they are clinically actionable. Progress will depend on larger, well-annotated cohorts using broad sequencing platforms, standardized reporting of variant subtype and co-mutation context, and longitudinal sampling across the disease course. Particular emphasis should be placed on relapse after targeted therapy, where BRAF and other MAPK-pathway lesions may reflect treatment-driven clonal evolution rather than baseline prognosis. In this setting, prospective genomic studies may help determine whether these alterations should influence translational trial design, resistance modeling, or the development of rational combination approaches. A key next step will be to move from descriptive detection of BRAF lesions toward defining their functional and therapeutic relevance in contemporary CLL.

##  Conclusion

BRAF alterations define a small but biologically meaningful subgroup of CLL. The most concise take-home points are that BRAF mutations are uncommon overall but recurrent; non-V600E variants predominate in typical CLL; clinically relevant signals often involve earlier treatment need or relapse biology rather than OS alone; and modern therapy-exposed cohorts support a role for BRAF within MAPK-pathway–mediated clonal evolution. At present, BRAF should be interpreted as a contextual biomarker within a broader genomic framework rather than as a routine standalone target, with the possible exception of transformed V600E-positive disease where pathway-directed therapy may merit individualized consideration.

## Key References

- Giménez et al. [12]. Important for defining the RAS–BRAF–MAPK–ERK subgroup in CLL, linking pathway mutations to adverse biologic features, and showing that treatment-timing endpoints are more informative than OS.
- Vendramini et al. [13]. Key study showing enrichment of KRAS/NRAS/BRAF lesions in trisomy 12 CLL and association with shorter treatment-free survival.
- Murali et al. [9]. Important mechanistic study linking MAPK-pathway activation to resistance to PI3K inhibition in relapsed CLL.
- Brown et al. [4]. Illustrates the emergence of acquired BRAF lesions in patients progressing on covalent BTK inhibitors in the ALPINE study.
- Sellar et al. [16]. Provides the main small-study signal that BRAF V600E may be enriched in Richter transformation relative to untransformed CLL.

## Data Availability

No datasets were generated or analysed during the current study.
